# The Future in Standards of Care for Gynecologic Laparoscopic Surgery to Improve Training and Education

**DOI:** 10.3390/jcm11082192

**Published:** 2022-04-14

**Authors:** Vlad I. Tica, Andrei A. Tica, Rudy L. De Wilde

**Affiliations:** 1 Department of Obstetrics and Gynecology, Doctoral School, University “Ovidius”—Constanta, University Emergency County Hospital of Constanta—Bul. Tomis, 140, Academy of Romanian Scientists, 900591 Constanta, Romania; vtica@eeirh.org; 2 Department of Pharmacology, University of Medicine and Pharmacy of Craiova, Emergency County Hospital of Craiova, Str. Tabaci, nb. 1, 200534 Craiova, Romania; 3 Pius Hospital, Carl von Ossietzky University, 26121 Oldenburg, Germany; rudy-leon.dewilde@pius-hospital.de

**Keywords:** laparoscopy, gynecology, standards of care, training

## Abstract

Standards of care offer doctors and patients the confidence that an established quality, evidence-based, care is provided, and represent a tool for optimal responding to the population’s needs. It is expected that they will increasingly express a multimodal relationship with gynecologic laparoscopy. Laparoscopy is, now, a standard procedure in operative gynecology, standards are embedded in many laparoscopic procedures, standardization of the skills/competency assessment has been progressively developed, and the proof of competency in laparoscopy may become a standard of care. A continuous development of surgical education includes standard equipment (that may bring value for future advance), standardized training, testing (and performance) assessment, educational process and outcome monitoring/evaluation, patients’ care, and protection, etc. Standards of care and training have a reciprocally sustaining relationship, as training is an essential component of standards of care while care is provided at higher standards after a structured training and as credentialing/certification reunites the two. It is envisaged that through development and implementation, the European wide standards of care in laparoscopic surgery (in close harmonization with personalized medicine) would lead to effective delivery of better clinical services and provide excellent training and education.

## 1. Why Standards of Care?

**Standards of Care** offer doctors and patients the confidence that an established quality of the service is provided. The Royal College of Obstetricians and Gynaecologists have described service standards as “standards of clinical care which the college would expect units and hospitals to adopt in relation to the quality of patient services, training opportunities and participation in national data gathering of relevance to clinical accountability and effectiveness” [1].

Evidence-based care is a solid and helpful concept, with influences not limited to the specialists’ activities, but also to the nurses’ ones, and is still debated at present [2].

Laparoscopy has shown in the last decades a steep evolution, including indications, procedures, increased patient comfort, advanced appropriate surgical skills, tools, and, beyond all this, a difference in the medical care paradigm. The changed dimension of the incision, or in the number of incisions (toward a single one) or, even, to the natural orifice surgery [3], depict only a facet of the progress. This evolution, in general, and particularly that of the gynecologic laparoscopy, was (and continues to be) prone to standards, which can improve the results of medical care.

## 2. Standards of Care; from Care to Training and Back

**Standards** are the criteria we can use to analyze quality. It is the institution’s responsibility to develop and to monitor standards, as a prerequisite in the process of pursuing higher quality. A diversity of standards responds to different needs, views, and organizations. Accreditation, ethics, client rights, billing, and professional/criminal records are only some examples.

Standards in laparoscopy may include criteria related to the equipment, facilities, surgical procedures, surgeons’ credentials/expertise, etc.

**Standards of Care** not only ensure that patients receive the best available care, but they are also a tool for better responding to the population’s needs. It is not possible, these days, to conceive such a system without including quality standards.

The application of standards should aim to obtain the expected outcome and to minimize risks for the population. Working with patients in the process of developing and implementing them would be a good option to obtain their partnership. Seeking beneficiary feedback and linkage to other existing programs represent another strategy in ensuring acceptance, support, and social integration.

As a procedure—but also as a philosophy—laparoscopy is the gold standard of care worldwide in the treatment of many abdominal pathologies such as appendicitis, cholecystitis [4,5,6], or even colorectal surgery. The faster recovery time, shorter hospitalization, decreased postoperative pain, faster return to daily activities, or cosmetic benefits are some of the advantages of laparoscopy [3].

**Standards of care and gynecologic laparoscopy** have a multimodal relationship.

1. First, laparoscopy is, now, a standard procedure in operative gynecology [7]. It became the standard of care for a great number of pathologies, such as management of adnexal masses [8], endometriosis (including ovarian endometriomas [9]), ovarian drilling [10], or tubal sterilization [11]. More recent evolutions prove the same for sacrocolpopexy in case of genital prolapse [12], hysterectomy [13] or myomectomy—with standards, also, for a very short hospitalization [14].

Laparoscopic urogynecology, in particular, showed relevant evolution and adaptation, especially after the FDA warning, allowing meshes to be used only for pelvic organ prolapse (POP) [15]. Laparoscopic sacropexy remains the gold standard for the surgical management of POP [16] but variants were developed [17]—as well as laparoscopic lateral suspension (LLS)—as a safe, optimal technique for apical and anterior POP treatment [16,18]. Prospective randomized controlled trials for LLS are lacking [19]. These developments add to the well-established laparoscopic Burch colposuspension [20].

Gynecologic oncology is another field where laparoscopy became the reference, and recommended, procedure. Endometrial cancer and pelvic lymphadenectomy represent such cases; cervical and ovarian cancer, and robotic-assisted surgery are areas with continuous assessment and development [21,22]. Cervical cancer is such an example: laparoscopic radical laparoscopy (LRH) was extensively developed for this pathology—even now, in a recent review, it seems associated with reduced preoperative morbidity, blood loss, and better postoperative recovery [23]. However, a large randomized trial [24]—supported, consecutively, by an epidemiologic cohort study [25] and a systematic review and meta-analysis [26]—reported an inferior outcome after LRH, compared with open surgery. The result was a significative drop in LRH [27], despite multiple articles published, subsequently, with controversial results [27,28]. Presently, LRH can be offered as standard of care to patients with early cervical cancer (IA2-IB1) [29]; specific recommendations were developed [30], while there is a need for further randomized trials [23,28].

**Recent developments** were reported. Laparoscopic minilaparoscopy, with shortened hospital stay and better esthetical outcomes [31,32], proved to be safe for benign adnexal surgery [33] or hysterectomy [34,35,36] while, in one study, inferior to standard laparoscopy in terms of operative time and surgical comfort [37]. Single-site laparoscopy (including robotic) was developed to reduce the number of skin incisions [38] and was reported to be safe for hysterectomy [35,36], while with a longer operative time and more postoperative pain than minilaparoscopy [35,36]. Microlaparoscopic procedures, which further reduce the size of the skin incisions, were recently used for salpingectomy [39], hysterectomy [40,41,42]—including for early endometrial cancer [43,44]—or colposacropexy [45]. Further studies are needed to assess these techniques’ advantages over standard laparoscopy [38]. Vaginal natural orifice transluminal endoscopic surgery (v-NOTES) was associated with better cosmetic results, reduced postoperative pain [46], and proved efficacious for ectopic pregnancy management [47], hysterectomy [46,48,49,50], or endometrial cancer surgery [51]. Laparoscopy could also exploit new energies, as lasers and plasma, which have been recommended for the surgical treatment of ovarian endometrioma [52], were proposed in the management of deep infiltrating endometriosis [53,54,55,56].

Another recent, unfortunate development, which profoundly influenced the laparoscopic surgery, was (and still is) the COVID-19 pandemic. Presently, “there is no consensus on limiting or restricting laparoscopic or robotic surgery” [57]—an assertion supported by scientific societies [58,59]—and the “consensus of surgical societies is to use a laparoscopic surgical approach for COVID-19 positive patients when appropriate” [60]. However, the laparoscopic option was significantly impacted by numerous reasons, such as resource limitation, surgeon reassignment to other appropriate COVID-19 tasks, reduction in beds allocated for such a surgery, reduction in elective procedures, protection against in-hospital viral spread, preoccupation for the security of the operating room (OR) staff, etc. [61,62]. Present recommendations favor postponing nonemergent or nonlife-threatening surgery for COVID-19 positive patients (including the ones with malignancy) until the infection is cured [58,59,60].

2. Second, standards are embedded in, now, a great number of laparoscopic procedures. In general surgery, a good recent example is represented by the laparoscopic right hemicolectomy [63]. In gynecologic laparoscopy, a suitable illustration is tubal sterilization [11]. These procedural standards influence surgical assessment/credentialing—depicted below. They represent the reference for alternative methods, such as ovarian mass extraction in endobag [64], or have, recently, been proposed for laparoscopic [65] or robotic [66] hysterectomy and for more difficult procedures, such as resection of deep infiltrating endometriosis [67]. Standardization of gynecological oncological laparoscopic procedures was considered imperative [68] and was successfully proposed for endometrial [66,69] or cervical [70] cancer. Another domain in which standards of care are considered is that of complications [71].

In urogynecology a search for better, easier, procedures, with better postoperative outcome, drove physicians to standardize modification of well-established procedures—such as the Burch colposuspension [72]—or to find alternative procedures, such as the previously mentioned LLS. Different modified techniques were proposed for LLS [73,74]—including robotic-assisted ones [75,76] as well as, recently, a standardized procedure [77]. Some procedures still wait for standardization in this field; various techniques are described for sacrocolpopexy, with little consensus for the technical aspects [78] and there is a certain heterogenicity among the relevant studies [79]. Further similar work is required in the standardization of the microlaparoscopy techniques [44], even if some standardization proposals have been recently made [41].

The COVID-19 pandemic imposed specific recommendations/standards related to the perioperative management for gynecologic laparoscopy, such as preoperative screening and testing, with subsequent preprocedure quarantine, online follow-up, and psychological support [58,59,60]. Even if only the possible transmission of HIV, hepatitis B virus, and Sabin poliomyelitis vaccine virus 2 [80]—not SARS-CoV-2 [81]—were reported during minimally invasive surgery, recommendations include filtration for peritoneum and smoke evacuation, and minimizing the use of energy devices and operative staff exposure, including special personal protection equipment [57,58,59,82,83].

Standardization is also visible in the sterilization or disinfection equipment, facilities, and related procedures (and training) [84]. This is observed within the surgical environment and equipment used in gynecologic laparoscopy—with clear cases for laser or robotics [66,85,86]. New developments will, probably, include artificial intelligence [87].

Standards of the laparoscopic procedures’ documentation are essential for both assessment of care quality and for research [88].

3. Third, standardization of the skills/competency assessment has been progressively developed. A structured assessment of laparoscopic assistant skills (SALAS) was developed to assess camera navigation and was validated for laparoscopic cholecystectomies [89]. The same process was seen in gynecologic laparoscopy [90]. The objective assessment may include cognitive skills, technical skills, and surgical performance. Judgment is performed through verified tests and metrics, computer-based simulators, and review of unedited video recordings [91]. The Global Operative Assessment of Laparoscopic Skills (GOALS)—reported as widely used [92]—measures depth perception, bimanual dexterity, efficiency, tissue handling (on a Likert Scale-based score) but does not express surgical or clinical judgement, like autonomy [93]. Fundamentals of Laparoscopic Surgery (FLS) is another nonspecific procedure web-based (education and) assessment tool in basic laparoscopic surgery, and is appropriate, also, for gynecology [94] (https://www.flsprogram.org, accessed on 13 February 2022). Developments on FLS were evaluated as a proficiency-standard in advanced laparoscopic surgery [95].

The Objective Structured Assessment of Technical Skills (OSATS) is considered, by some, as the standard for skills assessment [96]. An Objective Structured Assessment of Laparoscopy was developed, validated, and used for salpingectomy [97,98,99] and hysterectomy [65]. A Laparoscopic Skill Index was developed for the measurement of gynecologic laparoscopy proficiency [100].

4. Finally, the proof of competency in laparoscopy may become a standard of care [101]. Without any claim of historical relevance or exhaustiveness, such a system of credentialing was established, for advanced gynecologic endoscopy, by the Accreditation Council for Gynecologic Endoscopy (ACGE), in 2001 [102]. The International Society for Gynecologic Endoscopy (ISGE) promotes a certification system based on three steps, with specific embedded standards: bachelor, laparoscopic surgeon, and master [103]. Standards for surgical competencies are also issued by the American Association of Gynecologic Laparoscopists (AAGL)—for both laparoscopy [104] and for robotic surgery, generally [105]—but, apparently, not for experienced surgeons [106]. These standards can equally be used for assessing minimally invasive gynecologic surgery fellowships [107]. Under the auspices of the AAGL, such a fellowship in minimally invasive gynecologic surgery [108] is managed, with the appropriate requirements, by the respective board [109].

In Europe, such accreditation standards were implemented by the European Society for Human Reproduction and Embryology (ESHRE) in 1997, and are comprised of four levels of expertise [110]. A joint certification program—the Certification for Reproductive Endoscopic Surgeons (ECRES)—is currently being developed with the European Society for Gynaecological Endoscopy (ESGE) [111].

ESGE, in collaboration with the European Academy of Gynecological Surgery (EAGS), has also developed training models, structured training programs [112] and a certification program—the Gynaecological Endoscopic Surgical Education and Assessment (GESEA) [113,114]. GESEA has incremental levels [115,116]: a certificate of theoretical knowledge and practical psychomotor skills followed by surgical competence, at three incremental levels: “Bachelor in endoscopy”, “Minimally invasive gynaecological surgeon”, and “Master in laparoscopic pelvic surgery”. It has been validated by other important societies: European—ESRHE, EBCOG (European Board and College of Obstetrics and Gynaecology), ENTOG (European Network of Trainees in Obstetrics and Gynaecology)—or North American—ACOG (American College of Obstetricians and Gynecologists) and AAGL [117].

A French version of GOALS proved to measure trainers’ progress [118]. The Society of European Robotic Gynecological Surgery (SERGS) proposed a standard education curriculum in robotic gynecological laparoscopy [119].

As it could be easily deducted, skill standards and surgical competency express the outcome of training.

**Training** involves standards as an essential constituent. Educational requirements vary according to the level of training and to the category of personnel. These standards may be more flexible or rigorous, depending on the needs of the trainees, and the level of the curriculum. Education and assessment instruments should be adapted to training/practice in as many institutions as possible, to obtain standardization and broad application [120]. Generally, training standards include provider evaluation and partnership, educational process assessment, trainees’ opportunities and requirements, financing, educational outcome, patients’ care, and protection, etc. All standards should be regularly reviewed.

**Training in gynecologic laparoscopy** follows the same, general, pattern, and expresses, at the same time, noticeable particularities.

Different surveys reported an increased interest in a standardized curriculum in gynecologic laparoscopy [107,121], including gynecologic oncology [122].

Standardized training and education in this field was developed in many regions/countries of the world [14,110,123,124,125,126]. As pointed by Bjerrum F et al., “laparoscopic simulation has become a standard component of surgical training” [127]. Specific trainings address 3D laparoscopy [128,129], virtual reality (VR) simulation [130], advanced gynecologic laparoscopy [92,131,132,133,134,135], or robotic-assisted laparoscopic gynecological laparoscopy [22,106,119,136]. Besides a structured curriculum related to existing standards, such as the FLS [137,138], most programs include standardized conditions [139], a standardized box trainer [131,139] or pelvic model [132], as well as standardized (dry lab) exercises/tasks [128,140,141]. Some drills may be specific, such as camera navigation [89], visio-spatial tests [128], or suturing [131,142]. The same is accurate for proficiency criteria [91,119,125,131]. Some authors advocate training performed on human body donors as the gold standard of education [133,143].

One of the most comprehensive standardizations of endoscopic surgical training is offered by ESGE (and, with a lot of similarities, by ISGE), as presented previously. Its face and construct validity were reported [144]. GESEA includes a theoretical assessment, followed by specific psychomotor skills acquisition (box training, camera navigation, hand–eye coordination, bimanual coordination, and suturing) and by a clinical development (surgical tutorials, demonstrations, clinical training, fellowship, congress) [115,116].

Training was significantly influenced by recent available evidence, by medical policies, or by the context. After the LACC trial [24], fewer LRH were performed by fellows [27,145]. The FDA warning—allowing meshes to be used only for pelvic organ prolapse (POP)— was associated by their recommendation to obtain specialized training for each mesh insertion procedure [15]. The COVID-19 pandemic resulted in a reduced trainees’ exposure for a multitude of reasons: recommendation against training on COVID-19 + patients [58], trainees’ redeployment to necessitating COVID-19 units, elective procedures’ reduction, cancellation of teaching activities, congresses, surgical courses, etc. [146]. The new context determined educational board to revisit the training curricula and looked for alternative ways of training [146]. Different approaches were proposed, such as homemade simulation models, video games, smartphone applications, webinars, surgical videos, mental imagery [147], remote training on web-based platforms [148], or remote mentoring [149,150].

Standard equipment may bring value for research and development. An eloquent illustration is the box trainer. Reports on its use, as a standard component of the study, addressed various aspects, as the training on an open box [151], elaboration of new simulation tools [152], new camera navigation training model [153], value of the experts’ evaluation of advanced laparoscopic skills [142], translation of simulation skills into the operation room [131], as well as appraisal of 3D laparoscopy [154,155], VR simulation [156,157], or robotic assistance [137].

Standardized testing and assessment, validated rating scales, and procedure-specific checklists, are of utmost importance in developing and conducting training programs [90,141,158,159]. The above-mentioned GESEA includes skills testing and clinical appraisal [115]. The previously described GOALS (standard and modified—including the French version) scale proved to be, besides its use in certification, a valuable tool in programs’ validation [160] and trainees’ evaluation [118,161]. Similarly, OSATS has been seen as a standard for various skills assessment [96,132,162,163], including for VR simulation [153], or as a source for a derived assessment tool for total laparoscopic hysterectomy [164]. Scoring systems may also appeal to translational knowledge, such as the National Aeronautics and Space Administration Load Index [165]. Computerized models could be one of the future solutions for reliable and objective assessment [166].

Performance standards can be set by different approaches: laparoscopic experts considered as reference, contrasting groups method or Angoff method [167,168]. There is, still, a need for developing standard methods for generating those goals, at least if based on expert values [169].

The educational outcome represents, evidently, the result of the above-mentioned education. As a meta-analysis, though, its standardization was embedded in the previously mentioned structured training, equipment, testing, performances benchmarks, and credentialing. Acquisition of a required knowledge level [115], and standard level procedures [141,167], have been advocated. There is a clear option and tendency towards the standardization of the training programs, to be proficiency/performance-based, instead of time-based [91,125]. This has been advocated, similarly, for training programs in advanced laparoscopy [131], VR [130], or robotic [22,119] surgery. Of course, besides passing the examination [170] and, eventually, certification, the final desired outcome, is the translated competency on live patients, as developed below.

The educational process assessment is essential, in validation with the curriculum [99,126] or evaluation of the program’s value [171]. Related to the subject of this review, in the USA in 2014, most programs lacked standardization of theoretical or skills assessment [121], while a recent national Canadian survey (published in 2021) identified “a general interest in standardized training” [107]. Results of such assessments are useful, not only for the respective programs, but for the interested medical community. One nine-year survey study confirms this, by suggesting that more experienced trainees (gynecology included) perform better on the cognitive skills [170]. Another group was able to substantiate the validity of a specific, structured, virtual reality training program for laparoscopic hysterectomy [153]. Relative to VR, a systematic review of randomized trials concluded that there was a “substantial evidence (grade IA–IIB) to support the use of VR simulators in laparoscopic training” [130], while another systematic review suggested that, for laparoscopy training, “there is no clear evidence of the superiority of one tool or method over the others in skill acquisition”, with a clear preference for a structured curriculum—integrating theory, simulation, and live surgery [172].

It seems that there is a need for more research in this field [172].

Patients’ care and protection represents not only a training standard, but also, now, an ethical criterion. It was supported, in recent years, by the introduction/validation of simulation-based training, and, even more, by normalizing it as a necessary stage in a structured education, before OR surgical training [115,169]. Laparoscopic learning in experimental surgical teams proved to be safe for patients [173].

**Monitoring** is the process through which one verifies if standards are met [174]. Standards, therefore, enable the process of programs’ evaluation (both as a process and as proposed result or impact). Compliance to standards is verified periodically. A variety of standardized questionnaires, scales, and checklists may be used to monitor patients’ status, postoperatively [175].

Organizations use various methods for monitoring: process supervision, supervisory visits, provider surveys, trainees’ assessments and feedback, educational planning, case management, program reviews, etc. [176]. The European Society for Human Reproduction and Embryology (issued quite a long time ago by its Committee of Special Interest Group on Reproductive Surgery), states in its guidelines, that the requirements for maintaining accreditation is on a three-yearly basis, which encompasses participation in ESHRE-organized/approved educational events, a yearly endoscopic procedures list, ESHRE audit, and a list of papers/participation in (multi-center) studies [110].

## 3. How do Standards of Care and Training Link Together?

Standards of care and training have a reciprocally sustaining relationship.

**(a) Training is an essential component of standards of care,** to support highly complex training programs.

One of the fundamental concerns of EBCOG relates to issues around inequity concerning health service provision across Europe and its impact on the quality of training for our future generation of “doctors in training” to sustain high quality services in Europe. This is especially important as “European integration allows free movement of persons, services, capital and goods” [177]. Therefore, within standards of care, a separate set of standards to facilitate a uniform quality of training are also desired. The EBCOG has developed two volumes of standards of care: one in obstetrics and the second in gynecology, officially launched in the European Parliament in November 2014. In the gynecology volume, within standard no.24 (laparoscopic surgery), there is, as for all other standards, a distinct section on training standards, which each training unit should endeavor to adhere to. Such standards relate to the EBCOG logbook implementation in training, the necessity of a dry lab, official bodies’ formal training or experience recognition, and regular training in communication skills, breaking bad news, cultural/gender awareness, equality and diversity, and the safeguarding of vulnerable individuals [177].

A supplementary benefit for the patients is that the training program and resources are founded on evidence-based practice and on solid ethical principles.

**(b) Care is provided at higher standards after a structured training.**

As stated previously in this text, the most important outcome of training is safe and good surgical performance. Another important goal is the avoidance of—or finding solutions for—difficult situations [178]. The concept was also considered for complicated operative laparoscopy [124]. Better and safe operative performance was reported as a result of a structured learning program [179,180]. This benefit has already been reported in laparoscopic gynecology, when related to traditional OR training [124,181].

Standardized surgical skills assessment could be useful for residents and not necessarily for gynecologic surgeons in practice [90]. Different authors even proposed a certain number of procedures before reaching surgical competence [49,182].

**Simulation-based training** gained a lot of interest, in recent decades, even if box training tasks do not have the variability, complexity and environment of the real OR (advanced) surgery [142]. We depict the main reasons for this development below.

Besides increasing (basic and advanced) psychomotor laparoscopic skills in a dry lab [183], laparoscopic stimulation training was reported to significantly improve surgical performance in animals [118]— including laparoscopic skills [131]—as well as in humans [91,116,184], gynecologic laparoscopy included [172,185]. Clinical augmented performance was not restricted to basic surgery, but observed, also, for advanced procedures [92,132]. One group reported such improvement (for vaginal closure with prevention of prolapse and sacrocolpopexy) for beginners as well as for experienced surgeons [186], while another group suggested a longer-than-expected learning process, even for experienced surgeons, in case of laparoscopic single-site surgery [187]. A 3D system might offer advantages over 2D imaging [186]. Training based on human body donors seems to have a special place in training, with similar results [133]. One group reported that these basic and advanced acquired skills for gynecologists are unforgettable over time [172]. Another group’s publication suggests a significant improvement of surgical proficiency after regular laparoscopic trainer warm-up, before minor (adnexal) or major (hysterectomy) gynecologic laparoscopic surgery [140].

Those results were obtained when related to different simulation training tools. Box training was already mentioned. VR-based training was reported to help transfer/increase skills in gynecologic laparoscopic surgery in trainees/gynecologists with minimal surgical experience [130,188,189,190,191]. Such progress was not observed, though, when experienced operators were assessed for a basic procedure (laparoscopic salpingectomy for ectopic pregnancy) [189]. Different simulation methods proved to be efficacious. Even if a Cochrane review (2013) concluded that VR training resulted in better dry lab results for trainees with limited laparoscopic experience [192], it seems that, in real OR surgery, there is no proof of superiority of one method over the other [98,172], including the effect on advanced laparoscopic gynecologic surgery performance [92].

As a supplementary benefit, a structured training could enhance the quality of the OR performance by standardizing the respective procedure—as it was reported for the total laparoscopic hysterectomy [193].

No matter the simulation tool, many authors support the idea that, compared to conventional clinical training, simulation-based training is superior, in terms of OR surgical proficiency, for both basic [98,99,194,195] and advanced [92] gynecologic laparoscopies. A reasonable deduction would be that the traditional apprenticeship training is not adequate for the acquisition of the adapted surgical abilities [141].

However, surgical competence could depreciate over time when based exclusively on simulation training [172]. Another limitation is that, while novice and intermediate level gynecologists may benefit from this evidence-based data, expert surgical outcomes in advanced laparoscopy (hysterectomy) could not be related to the box trainer assessments [142]. These results are consistent with the report that experts do not improve anymore after repetitive exercises on the box trainer [196].

The best of the worlds resides in combining standardized, structured theoretical, simulation-based and live-surgery/OR training, as suggested by a recent systematic review [172].

**(c) Credentialing/certification**, mentioned previously, reunites the two directions of the standards of care–training relationship. One of the results is that certification and licensing (required by several countries or programs) are closely related to training requirements [174]. Specialty certification should only follow, for some authors, standardized skills assessment, prior to OR gynecology laparoscopy [121]. Such a structured training in laparoscopic gynecology may also be involved in hospital accreditation [123]. The mission is not easy, though, as we lack consistent studies on standard surgical competency criteria, related to skills tasks [197]—or their use in credentialing or maintaining certification [90]. There is a significant variability concerning surgical privileges; one such example is the higher proportion of community hospitals in the USA, compared with university centers, requiring preceptorship for laparoscopic hysterectomy or robotic sacrocolpopexy [198]. A very recent systematic review even concluded that there is a need for more research to defend “the use of FLS examination scores as a high-stake summative assessment” [138]. There are some promising research exceptions, such as the proposed tool for assessing competency in laparoscopic colorectal resections [199].

In the end, the most important accomplishment of training is both for patients and for providers: the certifying and continuously ensuring the fitness to practice.

It is envisaged that by implementation, the European wide standards of care, in close harmonization with the personalized medicine, would ultimately lead to effective delivery of not only clinical services but indeed provide excellent (standardized) training in these units.
